# Physical Activity Status and Position of Governmental Employees in Changing Stage Based on the Trans-Theoretical Model in Hamadan, Iran

**DOI:** 10.5539/gjhs.v7n5p23

**Published:** 2015-02-24

**Authors:** Jalal Abdi, Hassan Eftekhar, Mahmood Mahmoodi, Davood Shojayzadeh, Roya Sadeghi

**Affiliations:** 1 Department of Health Education and Promotion, School of Public Health, Tehran University of Medical Sciences, Tehran, Iran; 2 Department of Biostatics and Epidemiology, School of Public Health, Tehran University of Medical Sciences, Tehran, Iran

**Keywords:** physical activity, trans-theoretical model, employee

## Abstract

Physical inactivity is the fourth leading risk factor for death worldwide. Given the key role of employees as valuable human resources and increasing sedentary life style among them, the aim of this study was to evaluate physical activity status and position of governmental employees in changing stage based on the Trans-Theoretical Model (TTM) in Hamadan, Iran, in 2014. This descriptive-analytical study was performed on 1200 government employees selected using proportional stratified random sampling. Data collection was performed using a three-section questionnaire containing demographic characteristics, SQUASH (Short questionnaire to assess health-enhancing physical activity) questionnaire and Marcus et al’s five-part algoritm. Data were analyzed by multiple linear and logistic regression, Chi-square, T-test and ANOVA using SPSS-20.

The mean age of the participants was 38.12±8.04 years. About a half of the employees were in the preparatory stage of TTM.49.2% and 50.8% of the sample were classified as active and inactive, respectively. Associations between physical activity status and exercise stage of change were found. The associations between exercise stage of change and age, sex, work experience, education and marital status were significant (p<0.05). Work experience and education were strongly predictors of physical activity (PA) status and accounted for 31.2% of variance in PA (adjusted R^2^=0.312, R^2^ change=0.01).

The results of this study showed that TTM was useful to evaluate and predict physical activity behavior among the Iranian governmental employees and can be utilized by health planners to inform appropriate intervention strategies, specifically in work place.

## 1. Introduction

The World Health Organization (WHO) rates physical inactivity as one of the leading risk factors of death in the world. Physical inactivity levels are rising in many countries with major implications for the prevalence of non communicable diseases (NCDs) and the general health of the population worldwide. About 3.2 million people die due to lack of physical activity every year (WHO, 2009, 2010 & 2013).

With changing social and economic patterns all over the world, sedentary lifestyles have become a worldwide phenomenon (Lee, Macfarlane, Lam, & Stewart, 2011). This phenomenon is common in teenagers, adults, and elderly people worldwide (Timori & Esmailnasab, 2011).

Epidemiological evidence suggest that physical activity (PA) plays an important role in the prevention and reducing the risk of diseases such as cardiovascular disease (Juraschek et al., 2014; Porebska & Mazurek, 2014; Reed et al., 2014), diabetes (Chimen et al., 2012; Connelly, Kirk, Masthoff, & MacRury, 2013), cancer (Friedenreich et al., 2014; Santa Mina et al., 2014), and in weight management to prevent obesity (van Wier et al., 2006).

Physical inactivity is common in Iran (Esteghamati et al., 2011; Solhi, Ahmadi, Taghdisi, & Haghani, 2011; Hashemi, Rakhshani, Navidian, & Mosavi, 2013) particullary in employees (Jalilian, Darabi, Sharifirad, & Kakaei, 2013; Abdi, Eftekhar, Mahmoodi, Shojayzadeh, & Sadeghi, 2014) and a large number of people in Iran are not getting recommended physical activity and are deprived of its benefits (Saffari, Amini, Ardebili, Mahmoudi, & Sanaeinasab, 2012; Kaveh, Golij, Nazari, Mazloom, & Rezaeian Zadeh, 2014).

The work setting is as an important area of action for health promotion and disease prevention (Engbrers, 2008; van Wier et al., 2009). In work place physical activity can reduce the rate of absence from work and accordingly increases the productivity and engaging in job. Promoting mental health, self-esteem, mood, and reducing the risk of stress and depression are among the benefits of physical activity (Pirasteh et al., 2012; WHO, 2013; Strijk, Proper, van Mechelen, & van der Beek, 2013).

Theory-based studies to understand and promote physical activity behavior are more effective than atheoretical approaches (Glanz & Bishop, 2010; Plotnikoff, Lubans, Penfold, & Courneya, 2014). Knowledge about factors influencing physical activity behavior is needed in order to tailor physical activity interventions to the individuals (Sjors, Bonn, Trolle Lagerros, Sjolander, & Balter, 2014). The trans-theoretical model (TTM) has emerged as a frame work to understand, explain and predict how and when individuals initiate and adopt regular PA (Marcus & Simkin, 1994; Johnson et al., 2008; Paxton et al., 2008; Salehi, Eftekhar, Mohammad, Taghdisi, & Shojaeizadeh, 2010; Haakstad, Voldner, & Bo, 2013). This model proposes that individuals move through a temporal sequence of five stages: pre-contemplation (no intention of becoming regularly physically active), contemplation (intending to become regularly physically active within the next 6 months), preparation (intending to become regularly physically active within the next 30 days), action (being regularly physically active 30 min per day, most days of the week, but only within the last 6 months), and maintenance (meeting the requirements of PA for at least 6 months)(Johnson et al., 2008; Paxton et al., 2008).

Given the key role of employees as valuable human resources and increasing sedentary life style among them, the aim of this study was to evaluate physical activity status and position of governmental employees in changing stage based on the TTM in Hamadan, Iran.

## 2. Materials and Methods

### 2.1 Design, Participants and Setting

This cross-sectional, descriptive-analytical study was performed on 1200 employees working in the governmental sectors of Hamadan, west of Iran, in 2014.

### 2.2 Sampling and Data Collection

A total of 24551 employees were registered in the governor’s office list. According to the results of a prior study in Hamadan, Iran (Gharlipor, Sayarpor, & Moeini, 2011) with 80%power in a two-tailed tests at a significance level of 0.05, determined the sample size for the study at 1200.

The sampling strategy was based on a Proportionate stratified random sampling. We considered those governmental office with the members >50 who had agreed to participate in the study as one stratum. Considering the sample size, Proportionate allocation sampling was used to identify a sampling fraction for each office. The participants were randomly selected from the member’s list in each office. Participants were approached at workplace for a face to face interview by a trained interviewer.

After coordination with the directors of the offices, a questionnaire was distributed among the employees. The researchers provided the necessary information regarding the questionnaire completion and ethical issues including anonymity of the answers. Offices in this study were certain organizational units established according to the law with legal independence, executed part of the duties and responsibilities of one of the three executive, legislative, and judicial systems and other legal organizations.

### 2.3 Measurement Tools

Data were collected through a questionnaire with three sections demographic information, SQUASH (Wendel-Vos, Schuit, Saris, & Kromhout, 2003) and five parts algoritm (Marcus & Forsyth, 2003).

The SQUASH was developed in Netherlands to give an indication of the habitual activity level (Wendel-Vos et al., 2003).

The SQUASH refers to an average week in the past month and contains questions in the following domains: commuting activities, household activities, leisure-time and sports activities, and activities at work and school.

### 2.3.1 Calculating the Activity Score per Week from the Questionnaire

Activities were subdivided in to three intensity categories: 2 to <4 MET (light), 4 to 6.5 MET (moderate) and ≥6.5 MET (vigorous) with the help of Ainsworth’ compendium of physical activity (Ainsworth et al., 2000). Based on the reported effort in the questionnaire, activity scores per domain were calculated by multiplying the number of minutes per week with an intensity score (range 1 to 9) of the activities performed (Arends et al., 2013). The intensity score was based on the reported intensity of an activity combined with the activity intensity classification according to Ainsworth’s compendium of physical activities (Ainsworth et al., 2000) and Wendel et al.’s method (2003). The intensity scores are summarized in Table 1.

**Table 1:** Intensity score used for calculation of the SQUASH activity score

Intensity score based on reported effort			
	**Light**	**Moderate**	**Intense**
Commuting activities			
Walking to/from work or school	1	2	3
Bicycling to/from work or school	4	5	6
Leisure time activities			
Walking	1	2	3
Bicycling	4	5	6
Gardening	4	5	6
Odd jobs	1	2	3
Sports			
2 to<4	1	2	3
4 to 6.5MET	4	5	6
≥6.5	7	8	9
Household activities			
Light Household work		2	
Intense Household work		5	
Activity at work and school			
Light work		2	
Intense work		5	

The total score was calculated by talking the sum of the activity score for separate questions. based on calculated score participants were categorized as either inactive with a score of 599 or lower, and active if they had a score of 600 or more. this method recommended by Brown and Bauman (2000) and replicated by Jones et al. (2013).

To evaluate the reliability and validity of the questionnaire, it underwent primary translation and re-translation by the authors and a number of experts in the field of health education and health promotion. The experts were requested to evaluate the tool in terms of face validity, clarity, readability, and relevance. Test-retest was used to evaluate its reliability. The questionnaire was completed by 20 employees twice in a two-week interval and a reliability coefficient of 82% was obtained. The five-part algorithm of Marcus et al. (2003) was used to evaluate the stages of change. Its reliability and validity in Iran were confirmed in two studies by Jalillian et al. (2013) and Moeini, Jalilian, Hazavehei and Moghimbeigi (2012).

### 2.4 Data Analysis

Data were analyzed with SPSS-20 using descriptive and analytical statistics. Frequencies, percentage, mean and standard deviation were used to describe demographic characteristics. To assess differences in characteristics, we used chi-square test for categorical variables and ANOVA for continuous variables as appropriate.

Multinomial logistic regression analysis with PA status as the dependent variable and TTM stages as independent variables was used to predict PA status. Also multiple linear regression was used to determine demographic predicting factors of PA.

### 2.5 Ethical Considerations

Ethics committee approval was obtained from the Ethics Committee of Tehran University of Medical Science (ID: 9021108006). Informed consent was obtained prior to study enrollment.

## 3. Results

The mean age of the employees was 38.12±8.04 years. Males and females comprised 50.6% (n=607) and 49.4% (n=593) of the participants, respectively. The majority of the employees (55.6%) had bachelor’s degrees and 13.2% had higher degrees. The employees had a mean work experience of 14.15±8.52 years. Most of the employees (81.8%) were married and had 1.24 offspring on average. About a half of the employees were in the preparatory stage of TTM. 49.2% and 50.8% of the sample were classified as active and inactive, respectively. Mean and standard deviation (SD) of activities of participants are summarized in Table 2.

**Table 2:** Mean and standard deviation (SD) of activities in study subjects

Intensity score based on reported effort	Mean	SD
Commuting activities		
Walking to/from work or school	89.90	142
Bicycling to/from work or school	9.60	60.70
Leisure time activities		
Walking	155	255
Bicycling	11	104
Gardening	24	156
Odd jobs	169	23
Sports		
2 to<4		
4 to 6.5MET	92	218
≥6.5		
Household activities		
Light Household work	609	736
Intense Household work	157	289
Activity at work and school		
Light work	2114	872
Intense work	374	121

The status of employees in commuting and leisure time activities was not favorable. Only in light work activities the employees reported acceptable scores. Sports activities more than 6.5 MET were reported in none of the subjects. 70 percent of employees did not have vigorous physical activity.

The associations between demographic variables and stage of change are displayed on Table 3.

**Table 3:** The socio-demographic variables and stages of change in governmental employees

Demographic Variables	Pre-contemplation	Contemplation	Preparation	Action	Maintenance	P-value
Age						
18-29	18(9.8)	25(3.7)	65(35.5)	56(30.6)	19(10.4)	0.00
30-39	53(10.8)	61(12.4)	239(48.5)	118(23.9)	22(4.5)	
>40	71(13.5)	94(17.9)	286(54.6)	60(11.5)	13(2.5)	
Sex						
Male	91(64.1)	92(51.1)	301(51)	103(44)	20(37)	0.02
Female	51(35.9)	88(48.9)	289(49)	131(56)	34(63)	
Marital status						
Married	121(12.3)	146(14.9)	500(50.9)	180(18.3)	35(3.6)	0.03
Other	21(9.6)	34(15.6)	90(41.3)	54(24.8)	19(8.7)	
Education						
Under Diploma	38(17.4)	51(23.3)	104(47.5)	19(8.7)	7(3.2)	0.00
Diploma	15(9.6)	24(15.4)	79(50.6)	32(20.5)	6(3.8)	
University degree	89(10.8)	105(12.7)	407(49.3)	183(22.2)	41(5)	
Work experience						
<10	54(10.7)	67(13.3)	213(42.3)	136(27)	33(6.6)	0.01
11-20	41(11.1)	56(15.1)	199(53.6)	62(16.7)	13(3.5)	
>20	47(14.4)	57(17.5)	178(54.6)	36(11)	8(2.5)	

**Table 4:** Logistic regression analysis to predict physical activity status (0= Inactive, 1= Active)

Variables	β	Std.Error	Wald	P	OR	95%CI
Pre-contemplation	-.298	.322	.851	.35	.742	.394-1.396
Preparation	.015	.314	.002	0.00	1.44	.794-2.622
Action	.332	.287	1.339	0.00	1.39	.496-2.448
Maintenance	.367	.305	1.450	0.00	3.28	1.021-5.665

^a^ The reference category is: Pre-contemplation. (Chi-square=5.736, p<0.05).

The associations between exercise stage of change and age, sex, work experience, education and marital status were significant (p<0.05).

Assumption of linear and logistic regression were verified. Multiple regression analysis showed that education and work experience variables were strong predictors of PA and accounted for 31.2% of variance in PA (Table 5).

**Table 5:** Regression analysis of demographic variables in predicting physical activity in study subjects

	β	Std.Error	Beta	t	P
Age	0.004	0.004	0.058	0.992	0.03
Sex	0.155	0.029	0.155	5.316	0.00
Work experience	-0/006	0.003	-.106	-1.83	0.01
Marital status	-.046	0.036	-.038	-1.261	0.03
Education	0.046	0.12	0.114	3.813	0/00

Subjects assigned to the higher stages of change (preparation, action and maintenance) manifested significantly higher rate of PA.

Females were at higher stages of change (action and maintenance) than males with a significant correlation between the stages of change and sex. comparing PA Status with the exercise stage of change variable, the logistic regression analysis showed that stages three to five were significantly different from stage one (p<0.05). Participants who were in stage three (preparation), stage four (action) and five (maintenance) were respectively 1.44 (95%CI: 0.794-2.622), 1.39 (95%CI: 0.496-2.448) and 3.28 (95%CI: 1.021-5.665) times more likely to be categorized as being active compared to those on stage one.

Multiple linear regression analysis with stepwise method showed that level of education and work experience were the strongest predictors of physical activity. They explained of 31.2% variance of physical activity (adjusted R^2^=0.312, R^2^ change =0.01).

## 4. Discussion

The current study was conducted to evaluate the status of physical activity among governmental employees in Hamdan city and some associated factors using the Trans-Theoretical Model. In this study demographic factors including age, sex, work experience, marital status and education affected PA. The status of employees in commuting and leisure time activities was not favorable and 50.8% of the sample were classified as inactive.

Determination of the status of the individuals in terms of the stages of health related behaviors is an appropriate reflection of their attitude and performance; the results can provide basic information to design appropriate educational programs for the designated behavior and thus help to prevent the related diseases and enhancing health and well-being in the society (Mazlomi & Mohamadi, 2012). Physical activity is one of the most important item of the lifestyle; It has a very important role in the prevention of lifestyle related diseases and health promotion, and enhances psychological performance, increases friendly gatherings, improves night’s sleep, and is considered an appropriate recreation (Hekmatpou, Shamsi, & Zamani, 2012). Recent studies have shown that a small percentage of adults are engaged in recommended levels of physical activity (Malik, Blake, & Suggs, 2014). In our study, about half of the participants were at the preparation stage, which was compatible with the results of the studies by Mazlomi and Mohamadi (2012) and Solhi et al. (2011). In a study by Mohamadi and Mehri (2012), half of the participants were at the pre-action stage. Moeini et al. (2010 & 2012) also reported similar findings in two studies.

In current study the amount of physical activity in the area of commuting activites (using bicycle to go to work or bicycling in leisure time) was low (Table 2). Koohpayehzadeh et al. (2014) showed that the overall prevalence of physical inactivity in Iran was increased from 15% (2007) to 21.5% (2011). Over the 4 years, 56.4 %, 39.2 %, and 74.4% of participants were physically inactive at work, commuting and recreation, respectively. The reasons can be environmental factors, the distance to work or the ownership of a car or bicycle. In a study by Demunter et al. (2012) similar results have been found.

Our findings showed that the relationship between physical activity level and the stage of change is significant. And logistic regression showed that employees who were in the maintenance phase were likely to be classified as being active 3.28 times more than those who were in pre-contemplation phase. These findings is consistent with the study of Jones et al. (2013) and Haaskstad et al. (2013). In our study 24% of employees were in action or maintenance phase, while in Jones et al. (2013) study, 48.2 samples were in the action or maintenance phase and similar correlation has been reported by Fahrenwald and Walker (2003).

Previous studies have suggested that many demographic characteristics and lifestyle factors tend to be associated with the participation in PA, such as gender, age, and education level (S. S. Hui, G. P. Hui, & Xie, 2014). Levels of physical activity decrease with age (WHO, 2009). Brown and Bauman (2000) using the formula MET. Min found that only 55.4% of 44-30 years old women have enough physical activity. While in women less than 30 years the rate is 68.3%. In our study the relationship between age and physical activity was also significant. This finding was inconsistent with study that performed by Jones et al. (2013).

Education is one of the important determinant of physical activity behavior (Garber, Allsworth, Marcus, Hesser, & Lapane, 2008; Gaston & Cramp, 2011) and those who have higher education are physically more active than those who have a lower education level. In our study, regression analysis showed that education is one of the predictive variables of physical activity. In line with the findings of current study, McNeill et al. (2006) in a cross-sectional study showed education influenced adult’s PA, as well. In Didarlo et al. (2011) study, education significantly affected PA in women. In Haakstad et al. study (2013) the relationship between education and physical activity was not significant.

Gender has a strong independent effect of the health and disease status, selection of lifestyles and living habits (Baheiraei & Mirghafourvand, 2011). Studies have shown that women use more health services than men, but underreport their health status when compared to men (Bertakis, Azari, Helms, Callahan, & Robbins, 2000). In the current study Women were at higher stages of change (action and maintenance) than men with a significant correlation between the stages of change and sex. These results are congruent with the findings of Mohamadi et al. (2012).

In current study, work experience was one of the predictors of individuals as being physically active or inactive. It seems with an increase in work experience there would be physiological, psychological and lifestyle changes that occur in the lifestyle of people which increases inactivity and decreases the amount of physical activity. In a study which conducted by the authors on employees lifestyle, there was significant relationship between work experience and life style (Abdi et al., 2014).

Family and its composition are the key factors influencing health-related behaviors (Feizi, Hosseini, Ghiasvand, & Rabiei, 2011; Saffari et al., 2012), in this study, the relationship between marital status and physical activity was significant. This result inconsistent with the findings of Jones et al. (2013), but is in line with the results of the study thatperformed by Bell and Lee (2005).

In present study, about 70 percent of employees did not have vigorous physical activity and their moderate physical activity was also not desirable. In the study of Fayaz-bakhsh et al. (2011), 44.1% of the participants declared that they never exercise. Also in Pirzadeh and Sharifirad’s study (2011) 76% of the individuals had partly desirable physical activity. Limitations in time and place are the most important factors for lack of physical activity and weight management in employees (Van wier et al., 2006, 2009; Lees, Clarkr, Nigg, & Newman, 2005).

### 4.1 Conclusion

The results of this study showed that TTM was useful to evaluate and predict physical activity behavior among the Iranian governmental employees and can be utilized by health planners to inform appropriate intervention strategies, specifically in work place.

### 4.2 Limitations and Strong Points

One of the limitations of this study is that MET. mins formula may lead to overestimation of the amount of energy spent. However, in this study, questionnaire and self-report of the amount of physical activity level is used. Concrete measurements in this study due to time and financial constraints were not possible. The generalizability of the results of this study, because it is carried out among governmental employees and not among employees in other sectors, including the private sector is limited. The data of this study have been collected in a time frame and we should be cautious in the interpretation of causative relationships. The strengths of this study include its large sample size and being theory based.
